# Mammographically occult breast cancers detected with AI-based diagnosis supporting software: clinical and histopathologic characteristics

**DOI:** 10.1186/s13244-022-01183-x

**Published:** 2022-03-26

**Authors:** Hee Jeong Kim, Hak Hee Kim, Ki Hwan Kim, Woo Jung Choi, Eun Young Chae, Hee Jung Shin, Joo Hee Cha, Woo Hyun Shim

**Affiliations:** 1 Department of Radiology and Research Institute of Radiology, Asan Medical Center, University of Ulsan College of Medicine, 88, Olympic-ro 43-gil, Songpa-gu, Seoul, 05505 South Korea; 2 Lunit Inc., 15F, 27, Teheran-ro 2-gil, Gangnam-gu, Seoul, 06241 South Korea

**Keywords:** Artificial intelligence, Mammography, Breast neoplasms

## Abstract

**Background:**

To demonstrate the value of an artificial intelligence (AI) software in the detection of mammographically occult breast cancers and to determine the clinicopathologic patterns of the cancers additionally detected using the AI software.

**Methods:**

By retrospectively reviewing our institutional database (January 2017–September 2019), we identified women with mammographically occult breast cancers and analyzed their mammography with an AI software that provided a malignancy score (range 0–100; > 10 considered as positive). The hot spots in the AI report were compared with the US and MRI findings to determine if the cancers were correctly marked by the AI software. The clinicopathologic characteristics of the AI-detected cancers were analyzed and compared with those of undetected cancers.

**Results:**

Among the 1890 breast cancers, 6.8% (128/1890) were mammographically occult, among which 38.3% (49/128) had positive results in the AI analysis. Of them, 81.6% (40/49) were correctly marked by the AI software and determined as “AI-detected cancers.” As such, 31.3% (40/128) of mammographically occult breast cancers could be identified by the AI software. Of the AI-detected cancers, 97.5% were found in heterogeneously or extremely dense breasts, 52.5% were asymptomatic, 86.5% were invasive, and 29.7% had axillary lymph node metastasis. Compared with undetected cancers, the AI-detected cancers were more likely to be found in younger patients (*p* < 0.001), undergo neoadjuvant chemotherapy as well as mastectomy rather than breast-conserving operation (both *p* < 0.001), and accompany axillary lymph node metastasis (*p* = 0.003).

**Conclusions:**

AI conferred an added value in the detection of mammographically occult breast cancers.

## Key points

- AI algorithm derived from a large-scale image database may identify tumor signs commonly masked to the human eyes.
- AI software could correctly detect 31.3% of mammographically occult breast cancers (“AI-detected cancers”).
- AI-detected cancers were mostly found in dense breasts.
- In the present study data, AI-detected cancers were more commonly found in younger patients, had axillary lymph node metastasis, and required more intensive treatment compared with undetected cancers.

## Background

Mammography is a widely used screening tool for breast cancer in many countries and is effective in reducing breast cancer-related mortality [1–4]. However, its sensitivity is limited and the prevalence of mammographically occult breast cancers ranges from 9 to 30% [5–9]. Traditional computer-aided detection (CAD) systems that mark focal areas of increased density and microcalcifications have been developed since the 1990s for automatic detection and classification of breast lesions in mammography. However, CAD systems failed to show significant improvements in the screening performance or cost-effectiveness, mainly due to the large number of false-positive findings [1, 4, 9–11].

With the recent evolution of artificial intelligence (AI) algorithms, there is much interest in its use in radiology, which is largely based on image data that can be easily processed and analyzed by computers [4, 12–15]. In terms of breast imaging, previous studies have found that AI algorithms have high sensitivity and specificity for the detection of breast cancers [1, 4, 9, 10, 16–19]; moreover, AI algorithms improved the radiologists’ cancer detection rates [9, 20] and achieved higher performance level than did radiologists without an AI assistance by reducing the rate of false-positive and false-negative interpretations [1, 9, 21]. In addition, AI algorithms significantly reduced the workload of radiologists without decreases in performance [3, 21–23] and showed promise for obviating the need for double reading [21].

Considering that AI algorithms are based on the imaging biomarkers derived from large-scale image data rather than traditional human-designed features of breast cancer, we speculated that tumor signs commonly masked to the human eye may be identified by AI algorithms [10]. However, only few studies have focused on the performance of AI algorithms for the detection of mammographically occult breast cancers. One study assessed the effectiveness of an AI algorithm in the reduction in false-negative interpretation by comparing the specificities of clinical and AI assessments at the mammography level, but the possibility of false-positive assessments in the AI algorithm (i.e., high malignancy score generated in the wrong area) was not taken into account [3, 21].

The purpose of this study was to evaluate the added value of an AI-based diagnosis supporting software in the detection of mammographically occult breast cancers with consideration of correct localization and to determine the clinical and pathologic patterns of mammographically occult breast cancers that can be additionally detected using the AI software.

## Methods

### Study population

The institutional review board approved this retrospective study and waived the need for informed patient consent for the use of anonymized patient data.

By retrospectively reviewing our institutional database, we identified 5480 patients who were pathologically confirmed with breast cancer by ultrasound (US)-guided biopsy in either screening or diagnostic setting between January 2017 and September 2019. Among them, we excluded 3590 patients who only had mammography performed in other institutions due to the inconsistent quality of the images and those with a history of breast surgery or vacuum-assisted breast biopsy. Of the remaining 1890 patients, 128 patients were determined to have no visible evidence of breast cancer on mammography (i.e., mammographically occult breast cancer) according to a single reading by one of six breast-specialized radiologists with at least 7 years of experience. These readers were provided with the clinical information of the patients, as well as the findings of prior mammography and other imaging modalities when available.

The mammography results were analyzed with Lunit INSIGHT MMG, version 1.1.1.0 (Lunit Inc.), which is an AI-based diagnosis supporting software for detecting breast cancer in mammography. For cases with a positive result for malignancy in the AI analysis in at least one of four standard views (i.e., right and left craniocaudal [CC] and mediolateral oblique [MLO]), the location with the highest suspicion for malignancy marked by the AI software (i.e., hot spot) was compared with the US and magnetic resonance imaging (MRI) findings as well as pathologic reports by one radiologist (HJK) to confirm that the AI software had correctly detected the malignant lesions. As a result, only those in which the location of the breast cancer was correctly marked on the mammography by the AI software were classified into “AI-detected cancers,” while the remaining cases were classified into “undetected cancers.”

### Artificial intelligence software

The AI software used in this study (Lunit INSIGHT MMG) was developed based on deep convolutional neural networks and was trained on 170,230 mammograms acquired from 36,468 breast cancer cases and 133,762 healthy controls at five institutions in South Korea (January 2004–December 2016), USA (January 2000–December 2018), and the UK (January 2010–December 2018) by using the equipment from Siemens Healthineers, Hologic Inc., and GE Healthcare [9]. There was no overlap between the data used for the development of the software and those used for the present analysis.

The AI software used the four-view, full-field digital mammograms obtained using devices from one of the three different vendors (Siemens Healthineers; Hologic Inc.; GE Healthcare) for analysis. The software provides results for each mammography image (i.e., one of the four standard views) in terms of pixel-level malignancy scores depicted as a color-coded heatmap, and also by a representative malignancy score, which is the maximum value of the pixel-level scores. The malignancy scores of 0–100 represent the level of suspicion, where 100 represents the highest suspicion of malignancy. A case with a malignancy score higher than 10 in at least one of the four standard views was regarded to have a positive result for malignancy, which was a cutoff that achieved 90% sensitivity in the tuning dataset used for the development of the AI software [9]. In addition to analyses based on a single cutoff, the changes in the proportion of AI-detected cancers at different cutoffs were evaluated. The AI software did not consider prior mammograms for analyses.

### Pathological analysis

The final histopathologic results of the surgical specimens were reviewed to determine the histologic type, nuclear grade, histologic grade, molecular subtype based on immunohistochemical staining of estrogen receptor (ER), progesterone receptor (PR), human epidermal growth factor receptor (HER)-2, and Ki-67 proliferation, invasive tumor size, and axillary lymph node status. For the three patients who did not receive surgery in our institution, the histopathologic results of the biopsy specimens were used instead.

### Statistical analysis

Statistical analyses were performed using IBM SPSS Statistics for Windows, Version 23 (IBM Corp., Armonk, NY, USA). To identify the difference between the AI-detected cancers and undetected cancers in terms of clinical and pathologic characteristics, Pearson’s chi-squared test or Fisher’s exact test was used for categorical variables and Mann–Whitney *U* test was used for continuous variables. All statistical tests were two-tailed, and *p* values less than 0.05 were considered statistically significant.

## Results

### Performance of the AI software in the detection of mammographically occult breast cancers

**Fig. 1:**
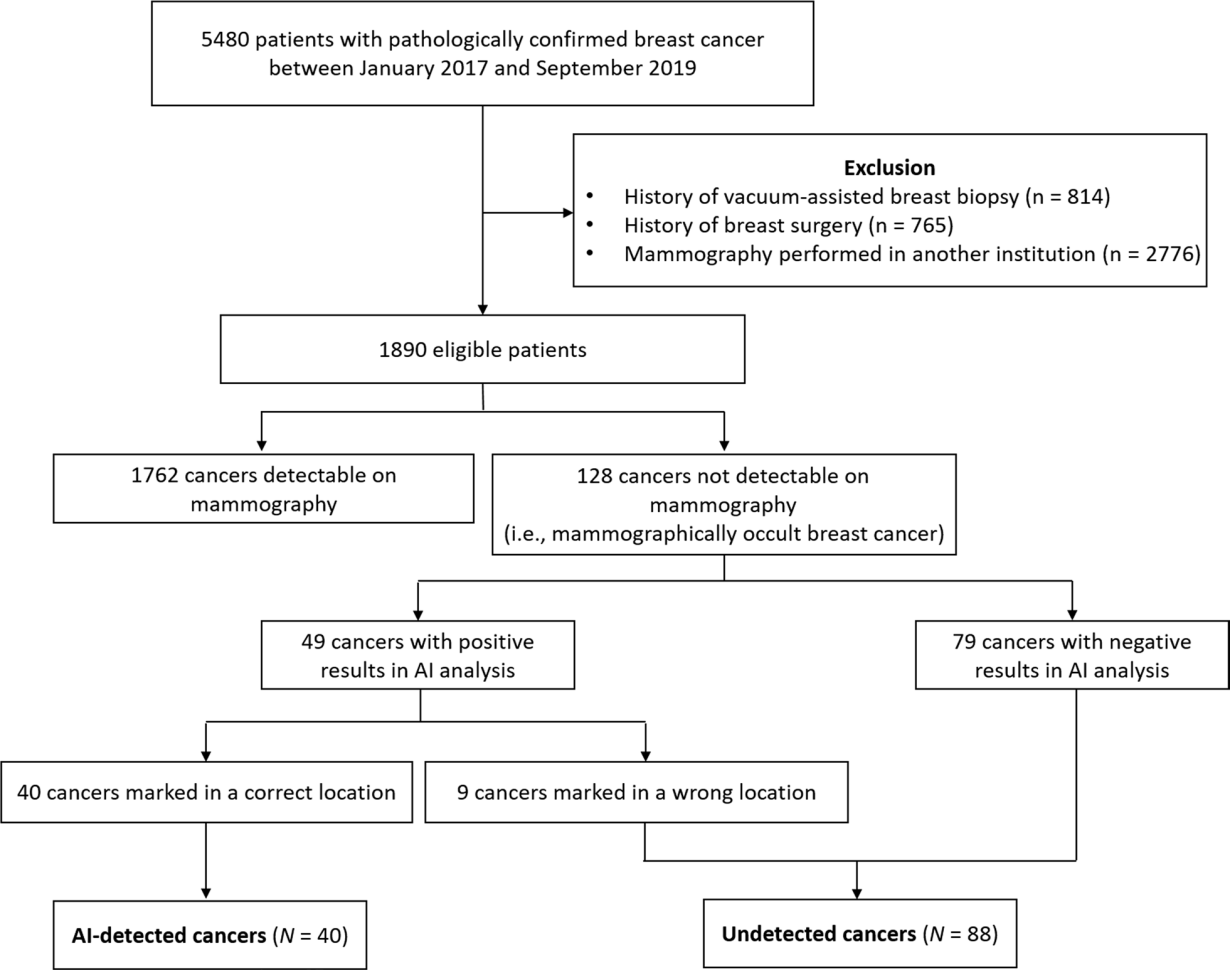
Study patient flowchart

In our study cases, mammographically occult breast cancers accounted for 6.8% (128/1890) of the total breast cancers. The AI software revealed positive results for malignancy in 38.3% (49/128) of the mammographically occult breast cancers in at least one of four standard views (i.e., right and left CC and MLO) at the cutoff score of 10. Among them, 81.6% (40/49) were marked in a correct location by the AI software, while the remaining 18.4% (9/49) had a high malignancy score in a wrong location. As such, at this cutoff, the AI software identified 31.3% (40/128) of mammographically occult breast cancers, and 2.1% (40/1890) of the total breast cancers were detectable only by the AI software based on mammographic interpretations. The results of the AI analysis of the 128 mammographically occult breast cancers are summarized in Fig. 1 and Table 1.

**Table 1:** AI analysis results of 128 mammographically occult breast cancers

	Mammographically occult breast cancer (*N* = 128)		
		Right	Left
AI-detected cancers	40 (31.3)		
Only on CC-view	3 (2.3)	1	2
Only on MLO-view	4 (3.1)	2	2
On both CC- and MLO-views	33 (25.8)	17	16
Undetected cancers	88 (68.7)		

Unless otherwise indicated, data represent the numbers of patients, with percentages in parentheses

*AI* artificial intelligence, *CC* craniocaudal, *MLO* mediolateral oblique

**Fig. 2:**
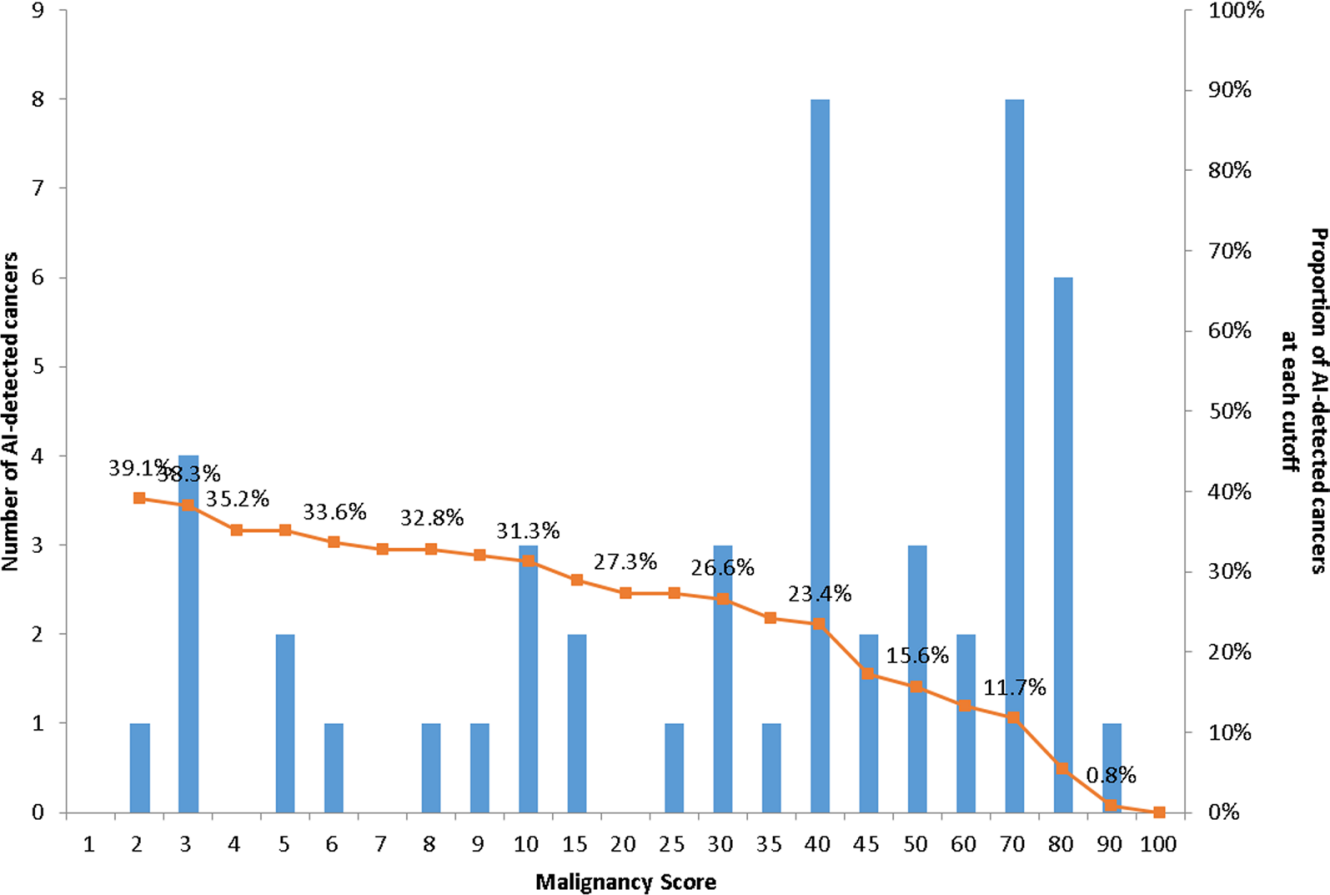
Proportion of AI-detected cancers according to different cutoffs. The proportions of AI-detected cancers among the 128 mammographically occult breast cancers at different cutoffs are shown as a line graph, with histograms displaying the distribution of malignancy scores.

The proportions of AI-detected cancers among the 128 mammographically occult breast cancers at different cutoffs are described with the histogram of malignancy scores in Fig. 2. Of note, the hot spot on the color-coded heatmap became less prominent in the lower malignancy scores and almost unidentifiable in malignancy scores of 2 or less; in other words, the proportion of AI-detected cancers could not be calculated for the cases in which the highest malignancy score of the four standard views was less than 2. When the cutoff was set to 2, 39.1% of the mammographically occult breast cancers could be correctly detected by the AI software.

### Clinical and pathologic characteristics of the mammographically occult breast cancers according to the result of the AI analysis

Among the 40 patients with AI-detected breast cancers (mean ± standard deviation [SD] age, 43.6 ± 9.8 years; range 25–66), the mean malignancy score was 53.6 (SD 24.1; range 11.2–94.7). Of them, 52.5% (21/40) were asymptomatic and diagnosed from screening US, while 47.5% (19/40) had palpable lumps in their breast. Four patients had a family history of breast cancer. Most of the patients had dense breasts, as 70.0% (28/40) had extremely dense breasts, 27.5% (11/40) had heterogeneously dense breasts, and only 2.5% (1/40) had scattered fibroglandular tissues. The tumors were almost evenly distributed in both sides of the breasts, and the most common location of the AI-detected tumors was the upper outer quadrant (UOQ) of both breasts (52.5%; 21/40). The mean size of the tumors measured in US was 17.0 ± 10.0 mm (range 4–52 mm). Breast MRI was performed in 33 patients, in whom all tumors except for one were detectable.

Regarding the histologic type of the AI-detected breast cancers, ductal carcinomas were in 85% (34/40) while 10% (4/40) were lobular, 2.5% (1/40) were mucinous, and 2.5% (1/40) were adenoid cystic carcinomas. The nuclear and histologic grades were 1 or 2 in 82.5% (33/40) and 81.1% (30/37) of the patients, respectively. The molecular subtypes of the 38 AI-detected breast cancer patients with available immunohistochemical profiles were luminal A in 55.3% (21/38), luminal B in 23.7% (9/38), HER-2 positive in 10.5% (4/38), and triple-negative in 10.5% (4/38).

**Fig. 3:**
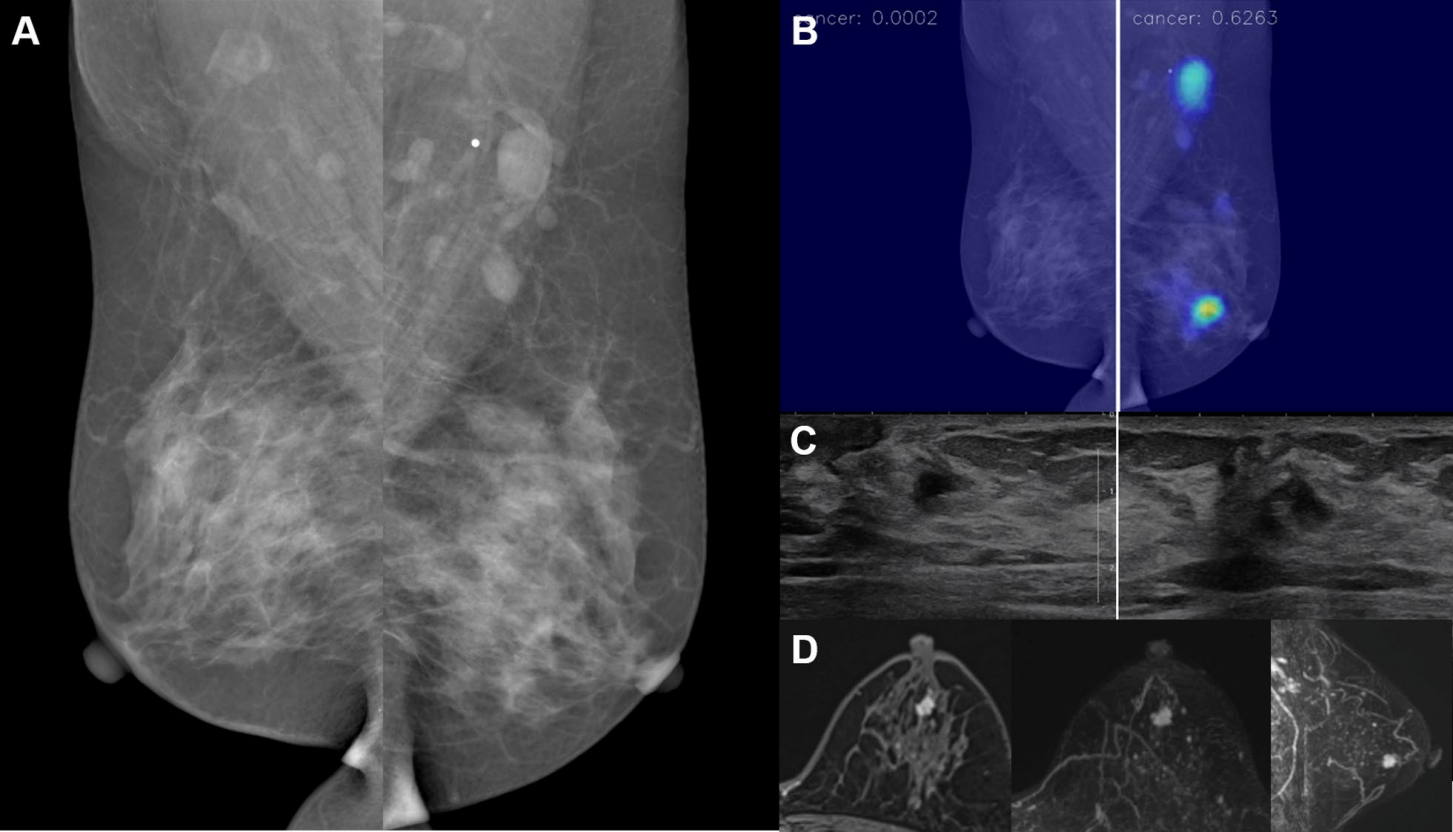
A 50-year-old woman with invasive ductal carcinoma in the left breast. **a** A BB marker was attached at the left axilla, where an enlarged lymph node was noted. However, no abnormal finding was detected in both breasts by a radiologist. **b**–**d** AI highlighted a focal area in the mid-portion of the left breast, which correlated with the location of the cancer in US and MRI. The malignancy score of the cancer was 62.6. Histopathologic examination of core needle biopsy and fine-needle aspiration showed a low histologic grade invasive ductal carcinoma that was estrogen receptor-positive, progesterone receptor-positive, and human epidermal growth factor receptor-2-negative, with axillary lymph node metastasis. Left central lumpectomy was performed after neoadjuvant chemotherapy, and no residual invasive ductal carcinoma was found

**Fig. 4:**
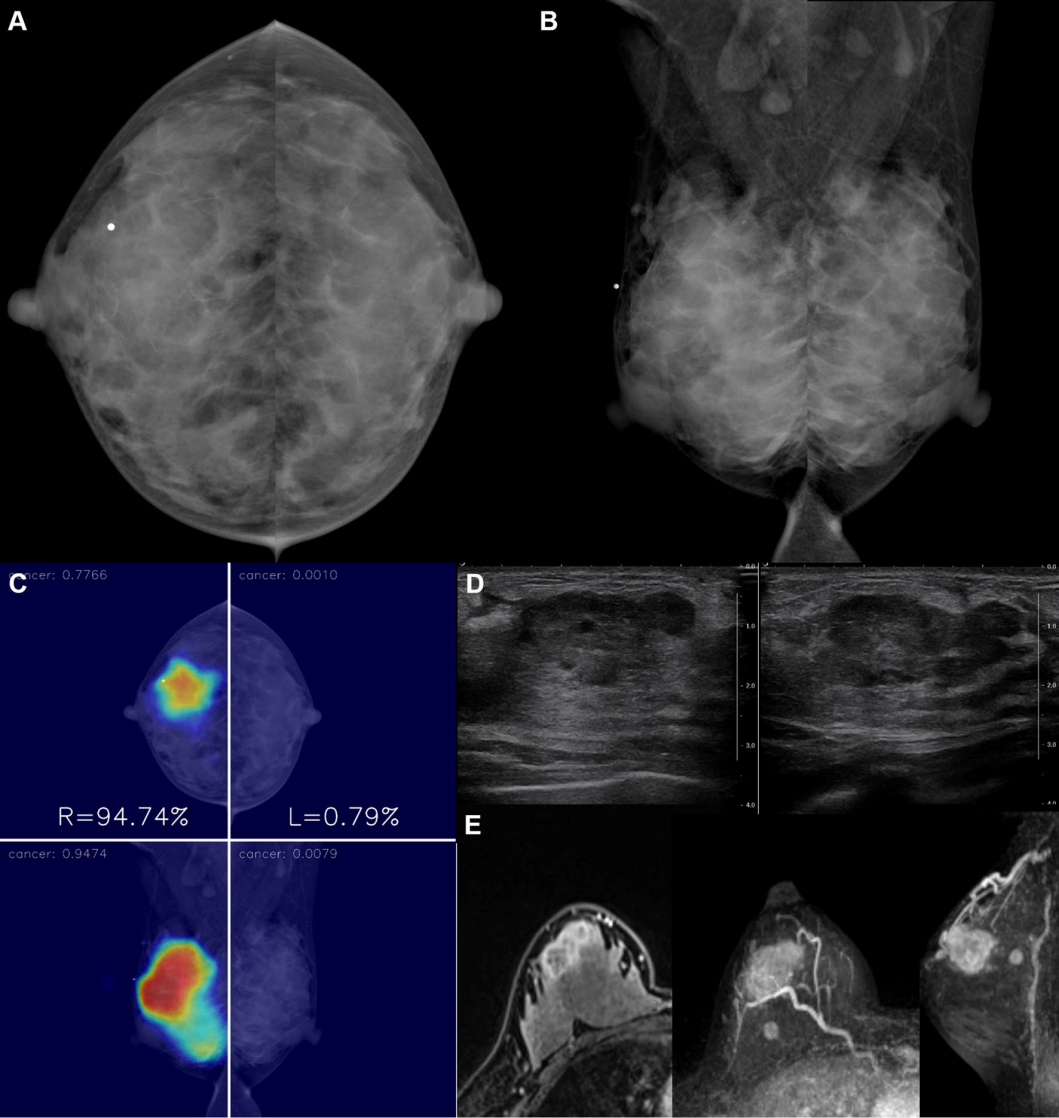
A 31-year-old woman with invasive ductal carcinoma in the right breast. **a**, **b** A BB marker was attached at the upper outer quadrant of the right breast, but no abnormal finding was detected in both breasts by a radiologist. **c**–**e** AI highlighted a focal area in the upper outer quadrant of the right breast, which correlated with the location of the cancer in US and MRI. The malignancy score of the cancer was 94.7. Histopathologic examination of core needle biopsy showed a high histologic grade invasive ductal carcinoma that was estrogen receptor-negative, progesterone receptor-negative, and human epidermal growth factor receptor-2-negative. Right mastectomy was performed after neoadjuvant chemotherapy, and a residual invasive ductal carcinoma was found

**Fig. 5:**
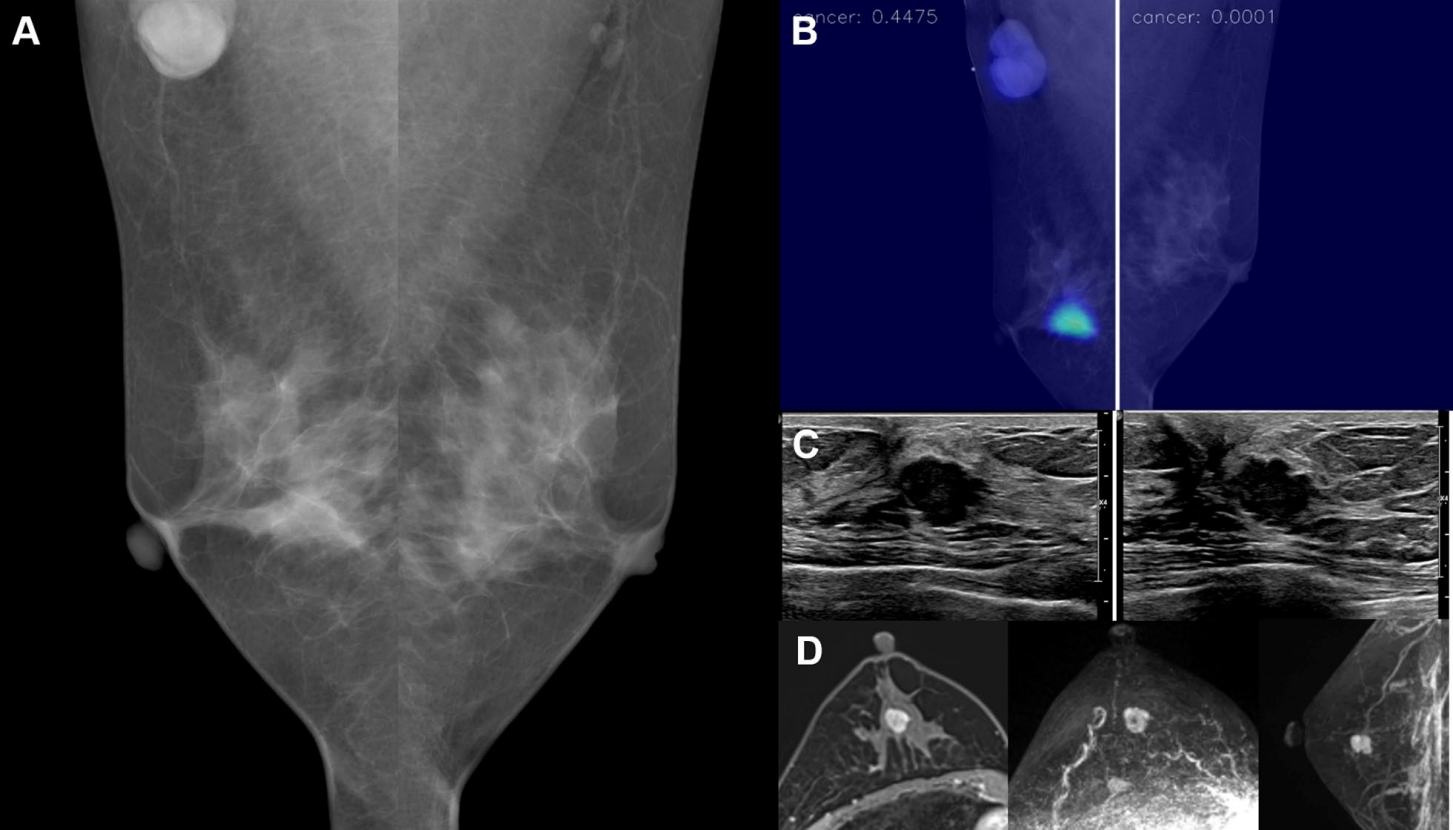
A 48-year-old woman with invasive ductal carcinoma in the right breast. **a** A BB marker was attached at the right axilla, where an enlarged lymph node was noted. However, no abnormal finding was detected in both breasts by a radiologist. **b**–**d** AI highlighted a focal area in the mid-portion of the right breast, which correlated with the location of the cancer in US and MRI. The malignancy score of the cancer was 44.8. Histopathologic examination of core needle biopsy showed a high histologic grade invasive ductal carcinoma that was estrogen receptor-negative, progesterone receptor-negative, and human epidermal growth factor receptor-2-negative. Right breast-conserving operation was performed after neoadjuvant chemotherapy, and no residual invasive ductal carcinoma was found

**Fig. 6:**
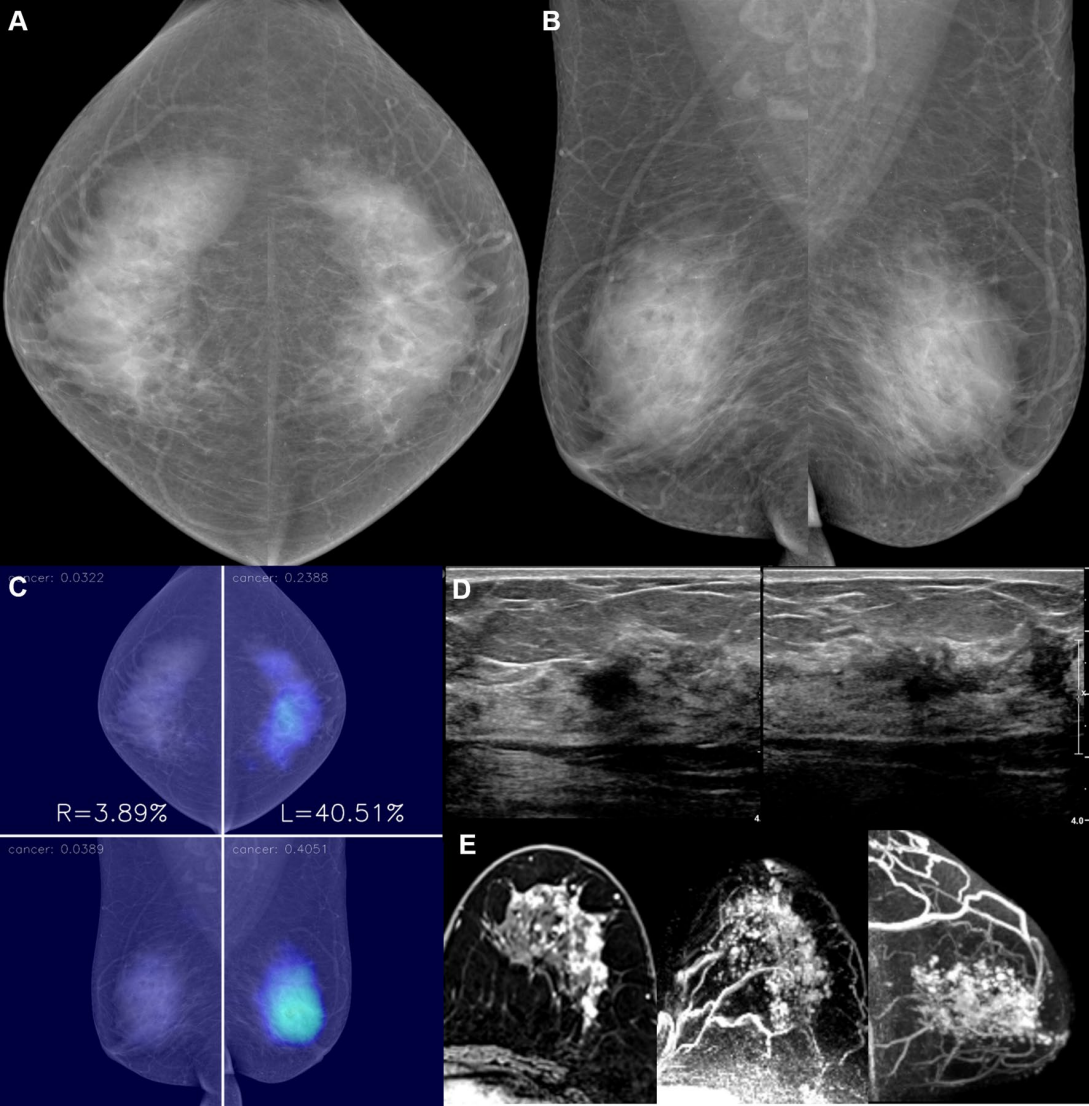
A 55-year-old woman with invasive lobular carcinoma in the left breast. **a**, **b**. No abnormal finding was detected in the mammography of both breasts by a radiologist. **c**–**e** AI diffusely highlighted the central portion of the left breast, which correlated with the location of the cancer in US and MRI. The malignancy score of the cancer was 23.9. Surgical histopathologic examination showed a node-positive, low histologic grade invasive lobular carcinoma that was estrogen receptor-positive, progesterone receptor-positive, and human epidermal growth factor receptor-2-negative by left lumpectomy

Of the 37 patients who were treated at our center, 32.4% (12/37) underwent neoadjuvant chemotherapy, and 56.8% (21/37) underwent a mastectomy while the remaining patients underwent a breast-conserving operation. There were invasive carcinomas in 91.9% (34/37), while the rest (8.1% [3/37]) were ductal carcinomas in situ, and there was a histologic upgrade in the surgical specimen in 16.2% (6/37) of the cases. The mean size of the invasive tumors that were not treated with neoadjuvant chemotherapy was 12.7 ± 14.1 mm (range 3–75). Axillary lymph node metastasis was present in 29.7% (11/37) of the patients. The example cases of AI-detected cancers are described in Figs. 3, 4, 5 and 6.

The mean age of the patients with AI-detected cancers was significantly younger than those with undetected cancers (*p* < 0.001). Patients with AI-detected cancers were more likely to be symptomatic and had a larger tumor size in US compared with those with undetected cancers (both *p* = 0.001). In terms of treatment, AI-detected cancers were more likely to undergo neoadjuvant chemotherapy as well as mastectomy rather than breast-conserving operation compared with undetected cancers (both *p* < 0.001). AI-detected cancers were more likely to be invasive carcinomas (*p* = 0.04) and had a higher likelihood of axillary lymph node metastasis (*p* = 0.003). In contrast, patients with AI-detected cancers were not significantly different from those with undetected cancers in terms of family history, mammographic breast density, lesion location, detectability on MRI, nuclear and histologic grade, molecular subtype, histologic upgrade rate, size of the invasive tumor, and lymphovascular invasion. The patient and tumor characteristics of the 128 mammographically occult breast cancers are summarized in Table 2.

**Table 2:** Characteristics of mammographically occult breast cancers according to the AI analysis

Variable	AI-detected	Undetected	*p*
Total	40 (31.2)	88 (68.8)	
Age^a^	43.6 ± 9.8	50.9 ± 9.3	< 0.001
Malignancy score^a^	53.6 ± 24.1	2.7 ± 6.2	< 0.001
Symptom			
Yes	19 (47.5)	16 (18.2)	0.001
Palpable lump	19 (47.5)	10 (11.4)	
Bloody nipple discharge	0 (0)	6 (6.8)	
Family history			0.37
Yes	4 (10.0)	14 (15.9)	
Breast density			0.19
A	0 (0)	1 (1.1)	
B	1 (2.5)	5 (5.7)	
C	11 (27.5)	38 (43.2)	
D	28 (70.0)	44 (50.0)	
Location (Laterality)			0.27
Right	21 (52.5)	37 (42.0)	
Left	19 (47.5)	51 (58.0)	
Location (Quadrant)			0.83
UOQ	21 (52.5)	47 (53.4)	
UIQ	9 (22.5)	24 (27.3)	
LOQ	5 (12.5)	7 (8.0)	
LIQ	5 (12.5)	10 (11.4)	
Ultrasonographic tumor size^a^	17.0 ± 10.0	13.4 ± 14.6	0.001
< 10 mm	8 (20.0)	43 (48.9)	
10–14 mm	13 (32.5)	28 (31.8)	
15–19 mm	6 (15.0)	9 (10.2)	
20–49 mm	12 (30.0)	3 (3.4)	
≥ 50 mm	1 (2.5)	5 (5.7)	
Detectability on MRI^b^			0.97
Yes	32 (97.0)	61 (96.8)	
Histologic type			0.45
Ductal carcinoma	34 (85.0)	79 (89.8)	
Lobular carcinoma	4 (10.0)	8 (9.1)	
Mucinous carcinoma	1 (2.5)	1 (1.1)	
Adenoid cystic carcinoma	1 (2.5)	0 (0)	
Nuclear grade			0.49
1	2 (5.0)	9 (10.2)	
2	31 (77.5)	67 (76.1)	
3	7 (17.5)	12 (13.6)	
Histologic grade^b^			0.51
1	3 (8.1)	10 (14.9)	
2	27 (73.0)	48 (71.6)	
3	7 (18.9)	9 (13.4)	
Molecular subtype^b^			0.59
Luminal A	21 (55.3)	54 (66.7)	
Luminal B	9 (23.7)	17 (21.0)	
HER-2	4 (10.5)	5 (6.2)	
Triple-negative	4 (10.5)	5 (6.2)	
Neoadjuvant chemotherapy^b^			< 0.001
Yes	12 (32.4)	5 (6.2)	
Operation method^b^			< 0.001
Mastectomy	21 (56.8)	18 (22.2)	
Breast-conserving operation	16 (43.2)	63 (77.8)	
Invasiveness^b^			0.04
DCIS	3 (8.1)	20 (24.7)	
Invasive carcinoma	34 (91.9)	61 (75.3)	
Histologic upgrade^b^			0.44
Yes	6 (16.2)	9 (11.1)	
Invasive tumor size^a,b^	12.7 ± 14.1	8.7 ± 9.5	0.93
< 10 mm	15 (40.5)	47 (58.0)	
10–14 mm	9 (24.3)	18 (22.2)	
15–19 mm	5 (13.5)	6 (7.4)	
20–49 mm	7 (18.9)	10 (12.3)	
≥ 50 mm	1 (2.7)	0 (0)	
Axillary lymph node metastasis^b^			0.003
Yes	11 (29.7)	7 (8.6)	

Unless indicated otherwise, data are numbers of patients with percentages in parentheses

*AI* artificial intelligence, *UOQ* upper outer quadrant, *UIQ* upper inner quadrant, *LOQ* lower outer quadrant, *LIQ* lower inner quadrant, *DCIS* ductal carcinoma in situ

^a^Values are the mean ± standard deviation

^b^Percentages are calculated based on the number of patients who had available data

## Discussion

In our present analysis, the AI software identified 31.3% of mammographically occult breast cancers and increased the cancer detection rate by 2.1% based on mammographic interpretations. The mean malignancy score of the AI-detected breast cancers was 53.6 (range 11.2–94.7). A recent study that used the same AI software used in this study reported that the malignancy score of 53.3 was the cutoff for the highest 2% scores and that the recommendation of supplemental modality at this cutoff could increase the cancer detection rate by 7.1% [3]. In our study, 15.6% of mammographically occult cancers were detectable with the AI software at the cutoff score of 53.3.

The rate of false-positive cases that revealed high malignancy scores in the wrong area was 18.4% at the cutoff score of 10. Among these false-positive cases, the hot spots were marked in (1) asymmetric glandular tissue (44.4%; 4/9), (2) one of the four views of mammography in which the grayscale was erroneously set to increase the overall brightness (22.2%; 2/9), (3) enlarged metastatic lymph node rather than the breast mass (11.1%; 1/9), and (4) normal glandular tissue with unidentifiable cause (22.2%; 2/9). These false-positive cases may not be a significant discouraging factor for the use of the AI software considering that false interpretation of the asymmetric glandular tissue is also a common pitfall in radiologists’ interpretations and that erroneous hot spots on the suboptimal images were easily dismissable.

The AI-detected breast cancers found in our study were commonly found in dense breasts. The application of AI softwares to routine mammography may allow patients with dense breasts to avoid taking additional risk, discomfort, or time for diagnosis with supplementary tools. While one study reported that AI software has lower sensitivity and specificity for dense breasts compared with those of human readers [24], other studies reported that the sensitivity of AI software was not significantly affected by the breast density [9, 10]. Furthermore, the performance of radiologists could be improved in dense breasts with the aid of AI, as also suggested from our results [9].

A previous study reported that AI showed good performance in the detection of early-stage cancers [9]. Likewise, we observed that the AI-detected breast cancers included 78.4% (29/37) of T1 cancers and 70.3% (26/37) of node-negative breast cancers. In addition, more than half (52.5%; 21/40) of the AI-detected breast cancers were found in asymptomatic women. These findings suggest the value of the AI software in the early detection of breast cancers and improvement of patient outcomes. On the other hand, compared with undetected cancers, AI-detected cancers were more likely to have axillary lymph node metastasis and more likely to require intensive treatment such as neoadjuvant chemotherapy or mastectomy. As such, the cancers detected by the AI software tended to require urgent clinical intervention.

The distribution of AI-detected breast cancers among various histologic and molecular subtypes was not notably different from those reported in general breast cancer [25–27]. This is in line with the finding of McKinney et al., in which the distribution of cancer type was not significantly different between AI-detected and radiologist-detected cancers [21]. In previous studies, invasive carcinomas were more likely to be mammographically occult compared with DCIS [5, 28], and most of the cancers detected only by an AI software were invasive cancers [21]. Another study also showed that the sensitivity of an AI software was higher in invasive cancers than in DCIS [10]. In our study, the proportion of DCIS among the AI-detected cancers was similar to that in the general breast cancers reported in a recent statistics [29].

The important strength of our study is that we focused on the performance of an AI software in the detection of mammographically occult breast cancers consecutively extracted from a large number of patients. In addition, the results provided by the AI software were verified with the imaging findings of other modalities and pathologic reports, thereby confirming that the AI software correctly localized the cancers.

There are several limitations to our study. First, the results are from a single-center data with only cancer-positive mammograms. A prospective study on the screening mammography of the general population is needed for a more generalizable result. Second, patients with a history of vacuum-assisted biopsy or breast surgery were excluded from the study because post-biopsy or postoperative changes such as architectural distortion could interfere with the result of AI analysis. Further research may be necessary to evaluate the effect of postoperative or post-biopsy changes on AI analysis. Third, the AI software in the present study was not built to consider prior mammograms in the analyses. As such, the usefulness of an AI software exploiting a more wide range of information that radiologists usually rely on during mammogram evaluation should be investigated in further studies in order to improve the quality of analyses.

## Conclusions

Our study shows that an AI-based diagnosis supporting software may confer an added value for the detection of mammographically occult breast cancer. A further prospective study with a screening cohort may validate the clinical applicability of AI-based diagnosis supporting softwares.

## Data Availability

The datasets used and/or analyzed during the current study are available from the corresponding author on reasonable request.

## Abbreviations

AI: Artificial intelligence
CAD: Computer-aided detection
CC: Craniocaudal
DCIS: Ductal carcinoma in situ
ER: Estrogen receptor
HER: Human epidermal growth factor receptor
LIQ: Lower inner quadrant
LOQ: Lower outer quadrant
MLO: Mediolateral oblique
PR: Progesterone receptor
SD: Standard deviation
UIQ: Upper inner quadrnat
UOQ: Upper outer quadrant
